# Economic evaluation of 2014 cholera outbreak in Ghana: a household cost analysis

**DOI:** 10.1186/s13561-017-0182-2

**Published:** 2017-12-04

**Authors:** Dziedzom Kwesi Awalime, Bernard Bright K. Davies-Teye, Linda A. Vanotoo, Nkechi S. Owoo, Edward Nketiah-Amponsah

**Affiliations:** 1 Economics Department, University of Ghana, Legon, P. O. Box LG 25, Accra, Ghana; 2 Ghana Health Service, Regional Health Directorate, P. O. Box 184, Accra, Greater Accra Region Ghana

**Keywords:** Cholera, HIA, LIA, Cost of illness, Household, Ghana

## Abstract

**Introduction:**

Ghana experienced its worst cholera outbreak in three decades in 2014. Evidence of cholera economic costs on affected households has been limited. This study aimed at determining economic costs on households affected by the  cholera outbreak in a Coastal Region of Ghana.

**Methods:**

Two districts; High and Low Incidence Areas (HIA and LIA) were selected in comparative cost analysis and disease impact on affected households assessed based on scientifically documented economic indicators. A total of 418 (282 HIA and 136 LIA) households that experienced at least one case of cholera infection were interviewed. Direct and indirect costs were estimated. Correlates of household’s cholera infection were estimated using Tobit Regression model in STATA 13.

**Results:**

Average direct cost to households in HIA amounted to USD 106.88, almost 2 folds higher than LIA (USD 62.02). Potential cost saving of an episode of cholera is USD 99,201.28 in LIA and raises almost 8 folds in HIA (USD 782,611.60). Households in lowest income category had the highest incidence of cholera (0.073) compared to other categories plus other factors were significant in explaining cholera incidence.

**Conclusions:**

The study showed considerable differences in HIA and LIA costs with higher household economic impact of cholera on the lowest income category. Results underscore the need for pragmatic policy interventions  to avert recurrent outbreaks and emphasis huge potential  cost saving with reducing  cholera cases.

## Background

Economic measurements of disease complement clinical and epidemiological approaches in disease burden assessment. Economic analyses seek to address a number of policy questions on consequences of disease or injury [1]. Economic measurements ultimately translate into cost-savings with reduction of adverse health effects.

Health ‘shocks’ such as unexpected health expenditures, reduced functional capacity and lost income and productivity are primary risk factors for health impoverishment [2, 3]. These factors pose a great burden on households which experience diseases such as cholera that presents symptoms only after patient is acutely ill.

“Cholera represents an estimated burden of 1.3 to 4.0 million cases, and 21,000 to 143,000 deaths per year worldwide” [4]. However, there could be as much as 100,000 to 120,000 cholera deaths every year but countries normally fail to report actual numbers due to fear of external economic implications on sectors like trade and tourism [4]. These numbers are corroborated by Ali et al. [5].

In parts of Ghana, cholera is now endemic and the country experiences outbreaks about every 5 years. In 2014, Ghana together with Nigeria and DR Congo reported 83% of all cases in Sub-Saharan Africa [6]. In that same year, Ghana experienced its worst outbreak in three decades reporting 28,944 cases including 243 deaths coming only second to Nigeria in infection rates [6, 7].

Research on cholera in Ghana has focused more on epidemiology of outbreaks and little emphasis on economic costs. Studies which identify socio-economic factors [8–10] are not detailed but provide mostly socio-economic linkages.

This study estimated comparative cholera costs in high and low incidence areas (HIA & LIA) plus correlates among cholera affected households. These provide empirical evidence to a lean literature on economic evaluations of cholera.

## Methods

The study used Cost-of-Illness approach by Rice [11] including WHO guidelines [1] for estimating economic consequences of disease and injury. This guided the assessment of household costs. Data on direct and indirect cost implications were collected using structured questionnaires.

### Study sites

The study was conducted in La-Dadekotopon and Shai-Osudoku districts within the Greater Accra Region. Historically, the region has become the epicenter for cholera outbreaks in the country. At the end of 2014, La-Dadekotopon and Shai-Osudoku documented 1907 and 315 cholera cases respectively. These districts ranked second and eighth respectively among the top ten districts that reported cases and where selected as high and low incidence districts respectively. Distinctive feature of these areas are that one is urban, highly polluted indigenes with mostly poor communities whilst the other is rural sparely populated mixed communities. These inherent differences help to understand the incidence and costs implications within these areas.

### Data and sampling

Patient data was obtained from the Ghana Health Service line list for cholera outbreak. This contained names, place of residence, sex, age, laboratory test result, outcome of treatment and the telephone contact of patient. GHS used this database in contact tracing of cases and this same tool was used in tracing patients to their households. For the purposes of this study, population was defined as all positive cases of cholera reported from a particular district. These formed the basis of inclusion criteria with all other households excluded for no documented cases at the health facilities.

Random sampling procedure was adopted in selecting 418 households; 282 and 136 from a HIA and LIA respectively. More specifically, households were randomly selected from patient population database using a calculated sampling interval to help answer the research objectives of this study. Data collection was primary through interviews. Patients who were untraceable where replaced by new ones who were sampled through the same random selection process, traced and interviewed. There was over 90% response rate and those who refuse to grant interview were also replaced. All questionnaires were retrieved and data entered for analysis.

## Cost estimation

### Direct costs

Direct costs included; first aid, cost of transportation for patient and caregiver, consultation fees, drugs purchased, laboratory cost, facility admission cost (hoteling cost), under-the-table payments (unofficial payments), feeding costs (special diet and water) and burial costs (supervised burial) in the event of death. An accounting process was followed where all costs attributing to various components were summed to obtain the disease cost. Costs were separated based on low and high incidence area costs.

## Indirect costs

The method adopted for measuring indirect cost (opportunity cost of ailment) was similar to that adopted by Sarker et al. [12]. This was done by computing the average household earnings as the base for determining the opportunity cost for the household. This average was then multiplied by the time component spent by the patient or caregiver for the time spent in travelling to and fro health facility, on admission and recovery after discharge. The time components include; travel time to facility, time spent at facility till discharge and work or school days lost after discharge. Waiting time with cholera treatment is reduced to zero because all cases brought into facilities are treated as emergencies and hence are not significant to this study. To ensure time loss estimates are not overweighed, during data collection only actively employed patients and caregivers were assumed to be losing productive hours and unemployed patients and caregivers time loss assumed to be zero.

### Cholera correlates

The study further examined relationship between cholera affected households using Tobit Regression model by observing the relevance of income categories and other household characteristics in relation to the proportion of household infection. Household’s characteristics were examined within this framework, testing which income groupings bore the greatest burden of the outbreak.

The empirical model is specified as:

$$ iC={\beta}_0+{\beta}_1 INC+{\beta}_2 SEX+{\beta}_3 MS+{\beta}_4 RHH+{\beta}_5 AGE+{\beta}_6 EDU+{\beta}_7 HI+{\beta}_8 DWS $$

*iC* = proportion of Household members Infected (number of infected persons divided by total household size).

*INC*= income category (categorical dummy; base: income above GH¢750; USD 234.38).

*SEX*= sex (dummy variable; base: male).

*MS*= marital status (dummy variable; base: married).

*RHH* = relationship with household head (categorical dummy; base: Other dependents); head of household, spouse, daughter or son.

*AG* = age (continuous variable).

*EDU* = education (categorical dummy; base: highest education (above Secondary); none, basic, secondary/technical/vocational.

*HI* = health insurance status (dummy variable; base: not insured).

*DWS* = drinking water source (categorical dummy; base: Inside Plumbing/Inside Standpipe); water vendor, neighbouring house, public standpipe and others.

## Results and discussion

**Fig. 1:**
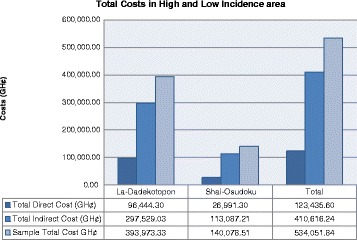
Total Costs in High and Low cholera incidence areas

The total direct cost incurred by households in the HIA and LIA amounted to GH¢96,444.30 (USD 30,138.84) and GH¢26,991.30 (USD 8434.78) respectively (see Fig. 1 and Table 1). Treatment costs in both high and low incidence districts formed the highest cost driver for households. When admissions costs were added, facility costs formed over 70% of all direct costs. These costs have important implications for the health system because during the outbreak, cholera treatment was declared free but this outcome shows the contrary. In the HIA, direct cost composition showed treatment cost (49.49%) as the largest component of the direct cost, followed by admission cost (23.47%), transportation for both patient and caregiver (12.63%), feeding (12.09%) then first aid (2.32%) (see Table 1).

**Table 1:** Direct costs in high and low cholera incidence area

Direct Cost	La-Dadekotopon		Shai-Osudoku		Total	
	GH¢	USD$	GH¢	USD$	GH¢	USD$
First Aid	2236.60	698.94	283.80	88.69	3219.34	1006.04
	(2.32)		(1.05)		(2.10)	
Treatment at Facility	47,729.00	14,915.31	12,885.00	4026.56	75,529.31	23,602.91
	(49.49)		(47.74)		(49.18)	
Feeding	11,658.00	3643.13	3684.00	1151.25	18,985.13	5932.85
	(12.09)		(13.65)		(12.36)	
Admission	22,637.00	7074.06	6470.00	2021.88	36,181.06	11,306.58
	(23.47)		(23.97)		(23.56)	
Transportation (Patient)	5912.00	1847.50	1410.00	440.63	9169.50	2865.47
	(6.13)		(5.22)		(5.97)	
Transportation (Caregiver)	6271.70	1959.91	2258.50	705.78	10,490.11	3278.16
	(6.50)		(8.37)		(6.83)	
Total	96,444.30	30,138.84	26,991.30	8434.78	123,435.60	38,573.63

Bracket figures are percentages

Exchange rate: 1USD = GH¢3.20 (Exchange rate as at December 31, 2014)

From Table 2 on average it cost a household in a HIA GH¢342.00 (USD 106.88) and GH¢198.47 (USD 62.02) in a LIA to seek treatment. When compared to average costs reported by Sarker et al. [12] in Bangladesh (USD 30.40) average costs are two folds higher in the LIA and more than three folds higher in the HIA.

**Table 2:** Individual and household direct average costs

	Individual Average Direct Cost				HH Average Direct Cost			
	La-Dadekotopon		Shai-Osudoku		La-Dadekotopon		Shai-Osudoku	
	GH¢	USD	GH¢	USD	GH¢	USD	GH¢	USD
First Aid	7.46	2.33	2.04	0.64	7.93	2.48	2.09	0.65
Treatment	159.10	49.72	92.70	28.97	169.25	52.89	94.74	29.61
Feeding	38.86	12.14	26.50	8.28	41.34	12.92	27.09	8.47
Admission	75.46	23.58	46.55	14.55	80.27	25.09	47.57	14.87
Transportation (Patient)	19.71	6.16	10.14	3.17	20.96	6.55	10.37	3.24
Transportation (Caregiver)	20.91	6.53	16.25	5.08	22.24	6.95	16.61	5.19
Total	321.48	100.46	194.18	60.68	342.00	106.88	198.47	62.02

*Exchange rate: 1USD = GH¢3.20 (Exchange rate as at December 31, 2014)

Source: Survey Data; Author’s computation from Excel

The average daily wage for households in the LIA was GH¢26.90, higher than in the HIA (GH¢22.80). On the other hand the average household size in LIA (3.6) is marginally smaller compared to HIA (3.7). These statistics have important bearings on the estimation of indirect costs within these two districts. Higher wages mean greater opportunity cost for lost man hours and household size influences the average household income; larger households mean lower per capita income and greater burden of the disease on that household.

**Fig. 2:**
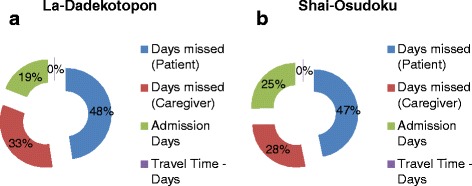
**a** and **b** Indirect Cost Composition in high and low incidence areas

In both districts, days missed by patients during recovery formed the largest composition of indirect costs but was 1% higher in HIA. Indirect costs associated with travel time were insignificant for both districts and is explained by existence of fairly easier access to transportation means in both districts (see Fig. 2). Average admission days was the same for both districts (3 days) but admission days formed a larger proportion of total indirect cost in the LIA (25%) than the HIA (19%) and in consonance, patients from LIA spent 2 days lesser away from normal daily activities than in the HIA (7 days). These suggest that complete recovery was faster and better in the LIA than the HIA.

From Table 3 an average of 25 days were missed in total by patients and caregivers away from their normal economic activities in the LIA but almost doubled in the HIA (48 days). This translated into GH¢1055.07 (USD 329.71) and GH¢831.52 (USD 259.85) average indirect cost for selected sample in the HIA and LIA respectively. Indirect costs in HIA were greater for all components than in LIA with the exception of productive days missed by caregivers. Total productivity loss by patients was GH¢ 141, 656.40 (USD 44,287.63) and GH¢ 52,858.50 (USD 16,518.28) in HIA and LIA respectively. That of caregivers was GH¢56,293.20 (USD 17,591.63) (HIA) and GH¢28,809.90 (USD 9003.09) (HIA). Together these costs formed over 70% of indirect cost composition.

**Table 3:** Days missed by patients and caregivers with indirect costs in high and low incidence areas

		Days missed (Patient)	Days missed (Caregiver)	Admission Days	Travel Time (Days)	Total Missed Days
La-Dadekopon		2071	1451	823	5	14,409
	*Average*	*6.90*	*4.84*	*2.74*	*0.02*	*48.03*
Shai-Osudoku		655	388	357	1	6816
	*Average*	*4.71*	*1.41*	*2.57*	*0.00*	*24.79*
	Total	2726	1839	1180	6	5751
		GH¢	GH¢	GH¢	GH¢	GH¢
La-Dadekopon	HH Total Indirect Cost	141,656.40	56,293.20	99,248.40	331.03	297,529.03
Shai-Osudoku	HH Total Indirect Cost	52,858.50	28,809.90	31,311.60	107.21	113,087.21
La-Dadekopon	HH Average Indirect Cost	502.33	199.62	351.94	1.17	1055.07
Shai-Osudoku	HH Average Indirect Cost	388.67	211.84	230.23	0.79	831.52
	Difference	113.66	−12.22	121.71	0.39	223.54

Source: Survey Data; Author’s computation from Excel

From Fig. 1 total cost to households were GH¢393,973.33 (USD 123,116.67) and GH¢140,078.51 (USD 43,774.53) for sample selected in HIA and LIA districts respectively. In HIA indirect costs was markedly greater (above GH¢200,000 (USD 62,500) more) but in LIA just slightly above GH¢100,000 (USD 31,250).

**Fig. 3:**
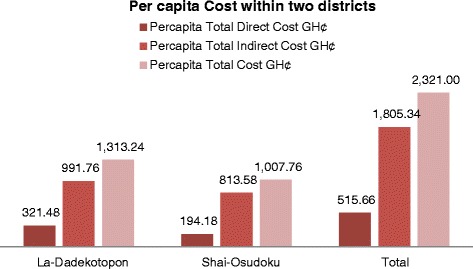
Total Cost of 2014’s Cholera Outbreak in High and Low Incidence Area

In total, 2222 cholera cases were reported in health facilities; HIA (1907) and LIA (315). When costs are projected for the total number of cases reported for both districts, the total cost of the 2014 cholera outbreak in a high incidence situation is GH¢2,504,357.12 (USD 782,611.60) and GH¢317,444.10 (USD 99,201.28) for lower incidence (see Fig. 3). Hence, if high incidence cases are reduced to levels of a lower incidence scenario, cost saving will be GH¢2,186,913.02 (USD 683,410.32).

**Fig. 4:**
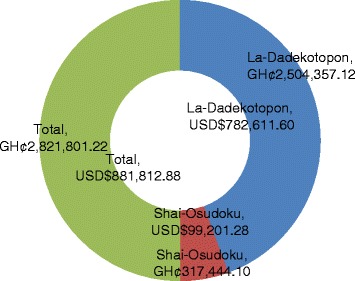
Per capita cost in High and Low incidence area

Per capita costs within these areas amounts to GH¢1313.24 (USD 410.39) in the HIA and falls to GH¢1007.76 (USD 314.93) in the LIA (see Fig. 4).

The regression result from Table 4 supports the fact that the impact of cholera is felt largest by the lower income categories. Compared to the highest income bracket, households in the least income bracket are 7% points more likely to experience a higher cholera incidence. For households within income brackets 3 and 4, there is a 6% points higher likely of infection compared with those in the highest income bracket. All are statistically significant at 10%. These results corroborate the percentages in the cross-tabulation on the infection rates among these income categories. Both Borroto & Martinez-Piedra [13] and Talavera & Pérez [14] studies support impacts of cholera being heaviest on the least income people.

**Table 4:** Tobit regression output showing coefficients, marginal effects for censored sample, standard errors and *p*-values

	Tobit Regression			Censored Sample		
Proportion of HH	Coef.	Std. Err.	P > t	dy/dx	Std. Err.	P > z
Income Grouping (Above GH¢750)						
C1: No Income	0.073**	0.032	0.022	0.019**	0.009	0.036
C2: Less than GH¢100	0.002	0.033	0.960	0.000	0.009	0.960
C3: >GH¢100 < GH¢350	0.059**	0.030	0.050	0.019**	0.009	0.037
C4: >GH¢350 < GH¢750	0.061**	0.030	0.042	0.019**	0.009	0.031
Sex (Male)						
Female	0.004	0.012	0.760	0.001	0.003	0.760
Marital Status (Married)						
Not married	0.137***	0.015	0.000	0.035***	0.004	0.000
Relationship with HH Head (Other Dependents)						
Head of HH	0.002	0.018	0.925	0.001	0.006	0.925
Spouse	−0.043*	0.022	0.054	−0.015*	0.008	0.059
Son/Daughter	0.146***	0.016	0.000	0.038***	0.004	0.000
Age	0.002***	0.000	0.000	0.001***	0.000	0.000
Education (Higher)						
None	0.008	0.036	0.820	0.002	0.009	0.822
Basic	0.017	0.034	0.619	0.005	0.009	0.610
Sec/Tec/Voc.	0.038	0.035	0.283	0.010	0.009	0.260
NHIS Enrolled (Yes)						
No	−0.015	0.011	0.167	−0.004	0.003	0.168
Drinking Water Source (Inside Plumbing/Standpipe)						
Water vendor	0.015	0.021	0.486	0.004	0.006	0.492
Neighbouring hse pipe	0.058***	0.016	0.000	0.016***	0.005	0.000
Public Standpipe	0.019	0.013	0.143	0.005	0.003	0.140
Other sources	0.004	0.027	0.879	0.001	0.007	0.880
Tobit regression		Number of obs = 1543				
		LR chi2(18) = 315.16				
		Prob > chi2 = 0.0000				
Log likelihood = −3801.5065		Pseudo R2 = 0.0398				

Significance levels = ***p < 0.01, **p < 0.05, *p < 0.1

Households with unmarried persons have 14% likely incidence than their married counterparts. In 2013, an Oxfam research report on gender and vulnerability to cholera in Sierra Leone showed higher infection among unmarried males compared to married males [15].

Within the household composition, the impact of cholera was highest among dependents of household heads in the sample. There was 15% higher cholera incidence among this group in relation to the head of household. Spouses however had 4% less likely incidence of the disease.

Among adults is a higher tendency to eat away from home and mostly from unregulated commercial food vender around their places of work. The regression shows that with an additional year in age of a household member, there is 0.2% points higher likelihood of cholera infection at 1% significance level. However, Deen et al. [16] in a study of three cholera endemic areas (Jakarta, Kolkata and Biera) found that in all three areas, the impact of the disease was highest among children under five. There seems to be some contextual underpinnings accounting for these mixed results from these two different studies. This can however be understood through future investigation.

Within household composition, cholera impact was greatest among household heads’ dependents. A 15% higher proportion of cholera incidence was among this group in compared to household head. Spouses however had 4% less incidence of the disease during the outbreak.

The incidence of cholera and sources of water cannot be exaggerated and studies such as Crooks & Hailegiorgis [17] support this fact. The safest source of drinking water as stipulated by the WHO is piped water on premises. Nketiah-Amponsah et al.’s [18] study’s the socioeconomic determinants of drinking water source in Ghana and found that income increases access to piped water in residence by 29 percentage points. Asante [19] also found a significant statistical relationship between income and access to safe or portable water. Based on these, inside plumbing and inside standpipe was set as the reference base for which other sources were compared. In the sample for this study, close to 70% of the households get their drinking water from a piped source, that is, an inside plumbing or in-house stand pipe (27.5%), tab water in neighbouring house (18.1) or public stand pipe (42.0%). These together formed 87.6% of drinking water sources that qualified as portable sources. The only water source that showed significance in relation to infection of cholera was water from neighbouring house. There was 5.8% higher cholera incidence in households that had their water sources from a neighbouring house as compared to household having inside plumbing as source of drinking water.

## Conclusion

Facility costs incurred by households formed the highest cost drivers (forming over 70% of all direct costs in both HIA and LIA) regardless of the government intervention of free treatment of cholera. HIA showed four times higher direct cost to households (GH¢96,444.30; USD 30,138.84) compared to LIA (GH¢26,991.30 or USD 8434.78), representing four folds increased cost when incidence rises from low to high incidence scenario. Average costs in these scenarios saw a 25% increase of costs in HIA households mostly resulting from increased out-of-pocket payments due to medical supply shortages in health facilities within HIA. In both districts, indirect costs were important and higher than direct costs. It was over 50% higher for both districts (51.0% and 61.4% higher in HIA and LIA respectively). In HIA, patients spent an additional 7 days in recovery after discharge from hospital but reduced to 5 days in the LIA. Total cost saving in averting an episode of cholera amounts to GH¢2,504,357.12 (USD 782,611.60) in HIA but rises 8 folds in a LIA scenario GH¢317,444.10 (USD 99,201.28). Factors such as income quintile, marital status, age and some drinking water sources were significant correlates with the incidence of cholera.

One limitation of this study is its assumption of unemployed patients or caregivers having zero indirect costs, but since illness and caring for the sick can prevent job hunting or accepting offers of work this is a limitation.

Also, not all sampled cases could be traced and interviewed so had to be replaced. This formed about 10% of all selected cases.

## Significance for public health

Cholera is a disease of poverty and continues to cause much strain on the resources of the health system as well as poor homes. The ease of spread and resulting costs cannot be downplayed. Cost analysis of cholera provides a key indicator of the financial strain on poor families when dealing with such menace.

- GHS must ensure the full and continuous implementation of its free cholera treatment.
- Need for social intervention policies such as free feeding or income compensations to mitigate impact.
- Importance of education on symptoms, first aid and need for early treatment by Information Services Department.
- Disparities of infection rates among different income groups plus other demographic variables highlights issues of discrimination and inequitable distribution of resources.

## Abbreviations

DHRC: Dodowa Health Research Centre
GHS: Ghana Health Service
GSS: Ghana Statistical Service
HIA: High incidence area
ISSER: Institute of Statistical Social and Economic Research
LIA: Low incidence area
WHO: World Health Organization

## References

[1] World Health Organization (2009). WHO guide to identifying the economic consequences of disease and injury.

[2] World Health Organization. The world health report 1999: making a difference. Geneva: World Health Organization; 1999.

[3] Xu K, Evans DB, Kawabata K, Zeramdini R, Klavus J, Murray CJ (2003). Household catastrophic health expenditure: a multicountry analysis. Lancet.

[4] World Health Organization. Weekly Epidemiological Record. 2015. Available from: http://www.who.int/wer/2015/wer9040.pdf?ua=1. [Accessed 24 Nov 2016].

[5] Ali M, Lopez AL, You Y, Kim YE, Sah B, Maskery B, Clemens J (2012). The global burden of cholera. Bull World Health Organ.

[6] World Health Organization. WHO Outbreak Bulletin. 2014;4(4). Available from: https://reliefweb.int/sites/reliefweb.int/files/resources/outbreak_bulletin_issue_4_-september_2014.pdf. [Accessed 24 Nov 2016].

[7] Ghana Health Service (2015). Report on cholera outbreak in the Greater Accra Region: June to December, 2014.

[8] Dotse E, Odoom JK, Opare JK, Davies-Teye BBK (2016). Outbreak of cholera, Greater Accra region Ghana 2014. J Sci Res Rep.

[9] Davies-Teye BB, Vanotoo L, Yabani JB, Kwaakye-Maclean C (2015). Socio-economic factors associated with cholera outbreak in Southern Ghana, 2012: a case-control study. Int J Epidemiol.

[10] De Magny GC, Cazelles B, Guégan JF (2006). Cholera threat to humans in Ghana is influenced by both global and regional climatic variability. EcoHealth.

[11] Rice DP (1966). Estimating the cost of illness (health economics series no. 6, PHS no. 947-6).

[12] Sarker AR, Islam Z, Khan IA, Saha A, Chowdhury F, Khan AI, Qadri F, Khan JAM (2013). Cost of illness for cholera in a high risk urban area in Bangladesh: an analysis from household perspective. Infect Dis.

[13] Borroto RJ, Martinez-Piedra R (2000). Geographical patterns of cholera in Mexico, 1991–1996. Int J Epidemiol.

[14] Talavera A, Perez EM (2009). Is cholera disease associated with poverty?. J Infect Dev Ctries.

[15] Rancourt N. Gender and Vulnerability to Cholera in Sierra Leone: Gender analysis of the 2012 cholera outbreak and an assessment of Oxfam's response. 2013.

[16] Deen JL, Von Seidlein L, Sur D, Agtini M, Lucas ME, Lopez AL, Kim DR, Ali M, Clemens JD (2008). The high burden of cholera in children: comparison of incidence from endemic areas in Asia and Africa. PLoS Negl Trop Dis.

[17] Crooks AT, Hailegiorgis AB (2014). An agent-based modeling approach applied to the spread of cholera. Environ Model Softw.

[18] Nketiah-Amponsah E, Aidam PW, Senadza B. Socio-economic determinants of sources of drinking water: some insight from Ghana. Conference on International Research on Food Security, Natural Resource Management and Rural Development, University of Hamburg; 2009.

[19] Asante FA. Economic analysis of decentralisation in rural Ghana. Peter Lang; Frankfurt am Main. 2003.

